# Death of Mrs. Potter

**Published:** 1906-12

**Authors:** 


### Death of Mrs. Potter.

EMILY BOSTWICK, wife of Dr. William Warren Potter, the editor of this JOURNAL, died at the family home, 284 Franklin Street, Buffalo, Wednesday morning, November 28, 1906, in the sixty-eighth year of her age. While she had been in indifferent health for several months the ending came somewhat suddenly, precipitated by cerebral apoplexy with which she was seized about fifty hours before her death. The services of the Protestant Episcopal Church were observed at the house on Friday, November 30, at three o'clock and the remains were incinerated at the Buffalo Crematory, the ashes being interred at Forest Lawn. The bearers were Dr. John O. Roe, of Rochester, and Drs. John Parmenter, Ernest Wende, H. E. Hayd, DeLancey Rochester, W. Scott Renner, William C. Krauss, and Lorenzo Burrows, of Buffalo.

---

THE POZZI "LIVRE D'OR,"—the souvenir volume presented to Professor S. Pozzi on the completion of 20 years of teaching at the Hospital Broca,—is said (by the *Revue de Gynecologie*) to mark an epoch in the history of gynecology in France. Pozzi's service was the first in France to assert the independence of gynecology and, in order to show the present standing of the science, Faure describes with minute detail an ordinary abdominal hysterectomy as done today in a well equipped operating room. The present record is that 95 per cent. of the patients are cured by the intervention, and he thinks we have reached the limit of perfection in our technic. The surgeons of A. D. 2000 will not perform an abdominal hysterectomy any better, he declares, than it is done today, although by means of discoveries beyond our ken at present they will not have to perform the operation so frequently as we do. All the articles in this number of the Revue are taken from the Pozzi souvenir volume.

---

DURING the recent meeting of the British Medical Association at Toronto, the Senate of Toronto University conferred the degree of LL.D. on the following members of the association and foreign guests: Dr. T. Clifford Allbutt, Dr. A. H. Freeland Barbour, Sir Thomas Barlow, Sir James Barr, Sir William H. Broadbent, Dr. H. W. Langley Browne, chairman of the council of the association; Mr. George Cooper Franklin, president of the association; Dr. William D. Halliburton, Sir Victor Horsley, Dr. Donald McAlister, president of the medical council of Great Britain; Dr. William J. Mickle, Dr. Louis Lapicque of Paris, Dr. L. Aschoff of Freiburg, Germany, and Dr. William J. Mayo, president of the American Medical Association. Four of the above were also recommended by the Board of Governors of McGill University for the degree of LL.D., these being Dr. T. C. Allbutt, Sir Thomas Barlow, Sir William Broadbent, and Sir Victor Horsley. A number of the recipients of the degree have already been similarly honored by one or more other universities.

---

AT the last meeting of the American Association for the Advancement of Science at Ithaca, June 30, 1906, a committee was appointed under resolutions of that body, to promote the establishment of a National Department of Health by agitation in all legitimate ways for the purpose of creating a public sentiment in its favor. The naming of the members of the committee is in the hands of Professor Irving Fisher, Yale University, chairman of this section of the American Association for the Advancement of Science. The names of the committee of one hundred selected will be announced shortly. The movement in general already has the support of the principal journals of medicine and hygiene in the United States.

